# Proteomics Reveals Mechanisms of Delayed Keratoconus Progression: A Study of Corneas Following Two Light-Activated Crosslinking Treatments

**DOI:** 10.1167/iovs.66.1.64

**Published:** 2025-01-30

**Authors:** Demi H. J. Vogels, Jurriaan Brekelmans, Ronny Mohren, Naomi R. N. Vos, Alexander Brandis, Arie L. Marcovich, Berta Cillero-Pastor, Avigdor Scherz, Vanessa L. S. LaPointe, Mor M. Dickman

**Affiliations:** 1 University Eye Clinic Maastricht, Maastricht University Medical Center, Maastricht, the Netherlands; 2 Department of Cell Biology–Inspired Tissue Engineering, MERLN Institute for Technology-Inspired Regenerative Medicine, Maastricht University, Maastricht, the Netherlands; 3 Department of Plants and Environmental Science, the Weizmann Institute of Science, Rehovot, Israel; 4 Maastricht Multimodal Molecular Imaging Institute (M4i), Maastricht University, Maastricht, the Netherlands; 5 Department of Life Sciences Core Facilities, the Weizmann Institute of Science, Rehovot, Israel; 6 Department of Ophthalmology, Kaplan Medical Center, Rehovot, Israel; 7 Faculty of Medicine, Hebrew University of Jerusalem, Israel

**Keywords:** crosslinking, cornea, proteomics, keratoconus, extracellular matrix

## Abstract

**Purpose:**

This study aims to elucidate on changes in biological pathways in rabbit corneas induced by two methods of light-activated corneal stiffening: topical application of riboflavin with dextran (RF-D) or WST11 with dextran (WST-D) followed by ultraviolet A (UVA) or near-infrared (NIR) illumination, respectively.

**Methods:**

Rabbit corneas were mechanically de-epithelialized, then left untreated (*N* = 3) or treated with either RF-D/UVA (*N* = 3) or WST-D/NIR (*N* = 3). After one week, quantitative proteomics was performed on untreated, RF-D/UVA– and WST-D/NIR–treated corneas. Pathway enrichment analysis was performed to identify the biological processes associated with the treatments. To identify the abundance and spatial distribution of lipids in the untreated, WST-D/NIR– and RF-D/UVA–treated corneal stroma, lipid mass spectrometry imaging was performed together with hematoxylin and eosin staining.

**Results:**

Between RF-D/UVA– and WST-D/NIR–treated corneas, 37 and 39 proteins, respectively, were differentially expressed compared to untreated corneas (*P* < 0.05). Pathway enrichment analysis showed the effect of RF-D/UVA treatment on cell metabolism and terminal differentiation of keratocytes, while WST-D/NIR modified extracellular matrix regulation and the mitogen-activated protein kinase signaling cascade. When comparing the RF-D/UVA and WST-D/NIR treatment, 74 proteins were differentially expressed, affecting cellular metabolism and respiration, complement activation, the activation of matrix metalloproteinases, and lipoprotein metabolism. The lipid profile for the RF-D/UVA– and WST-D/NIR–treated stromas were similar, whereas differences were observed comparing both treatments to untreated corneal stroma.

**Conclusions:**

Proteomics indicated a metabolic shift from oxidative phosphorylation to glycolysis and hypoxia after RF-D/UVA treatment. In contrast, WST-D/NIR stiffening maintained normal respiration and involved extracellular matrix remodeling.

Keratoconus is a bilateral, often asymmetric, ectasia of the cornea, causing progressive thinning, protrusion, and irregular astigmatism. The pathophysiology of keratoconus has not yet been fully elucidated, but is shown to be influenced by both genetic and environmental risk factors.^1^ Progressive keratoconus results in loss of visual acuity and, if untreated, could lead to extensive visual impairment. Given the onset is often during puberty, keratoconus is associated with a high personal and societal burden.^2^^–^^5^

In 2003, Wollensak et al.^6^ introduced corneal collagen crosslinking as a novel treatment to arrest keratoconus progression. In corneal collagen crosslinking, a photosensitizer is applied to the cornea and, on activation by specific light irradiation, induces crosslinking either directly through fibril bonding or indirectly through proteoglycans and thus arrests ectasia progression. Currently, the only clinically available corneal collagen crosslinking modality uses the chromophore riboflavin (RF) with subsequent irradiation by ultraviolet A (UVA) light. Although keratoconus progression has been arrested in more than 90% of the patients treated, there are several limitations of RF/UVA crosslinking, including the potential need to repeat the procedure, the recently recognized need for corneal oxygenation during treatment,^7^ and the concerning safety profile caused by the cytotoxicity of UVA irradiation, limiting the treatment of thin corneas.^8^^,^^9^

To overcome the limitations associated with RF/UVA crosslinking, several other stiffening modalities have been investigated. One of these alternatives comprises the application of a water-soluble bacteriochlorophyll photosensitizer, WST11, followed by near-infrared (NIR) illumination. WST11 was originally designed for vascular-targeted photodynamic therapy of solid tumors and is already authorized for prostate cancer treatment.^10^ Its topical application to rabbit corneas followed by NIR illumination results in effective and safe long-term stiffening.^11^ The efficacy of WST/NIR treatment has been confirmed to be similar to RF/UVA crosslinking, shown by a similar increase in resistance to enzymatic digestion,^12^ as well as comparable ultimate stress and Young's modulus after both treatments.^11^ No morphologic damage was observed in the posterior stroma or endothelium of corneas treated with WST-D/NIR, whereas severe endothelial damage was reported after treatment with RF-D/UVA in rabbits.^13^ In contrast to UVA light, NIR light is non-toxic to ocular structures at the applied intensities.^11^ The safety of the WST-D/NIR treatment is further ensured by supplementing the agent with dextran T500 (WST-D) to limit its penetration depth in the cornea.

The pathways induced by the RF-D/UVA crosslinking and WST-D/NIR treatment, which may halt keratoconus progression, have not yet been fully elucidated. Moreover, it is unknown whether any biological effects induced by these treatments contribute to the increase in biomechanical strength. This is the first study to compare RF-D/UVA crosslinking and WST-D/NIR treatment using both proteomics and lipidomics, offering novel insights into their underlying mechanisms and providing a comprehensive comparative analysis. Providing insights into the biological effects induced by both treatments gives more insight into halting keratoconus progression and the potential development of targeted therapies, particularly for optimizing safety and efficacy. Investigating protein changes is an important mean for unraveling signaling pathways that might underlie the halt in keratoconus progression through corneal collagen stiffening. Additionally, exploring the spatial distribution of lipids after stiffening is important as it provides valuable insight into the localization of the lipids within the cornea, next to their abundance. In this study, we aimed to investigate the biological pathways induced by both treatments and assess potential differences that may shed light on the yet unresolved mechanism of cornea stiffening upon photodynamic therapy. We used proteomics and mass spectrometry imaging (MSI) to investigate the pathways and the abundance and spatial distribution of lipids in WST-D/NIR– and RF-D/UVA–treated and untreated corneas in rabbits. This research offers a unique opportunity to correlate the biological changes induced by the treatments with clinically relevant effects, which could guide optimal treatment selection to allow personalized treatment or motivate the use of these treatments in other complications.

## Material and Methods

### Chromophores

Two chromophore formulations were prepared in saline solution: (1) 0.25% (w/v) WST11 (Steba Laboratories Ltd., Rehovot, Israel), and (2) 0.1% (w/v) riboflavin 5′-monophosphate (RF; Sigma-Aldrich, St. Louis, MO, USA). Both formulations were enriched by 20% (w/v) dextran 500 (from *Leuconostoc* spp. M_r_ 450,000–650,000; Sigma-Aldrich) and corrected to a pH of 7.2–7.3.

### Animal Model

Nine corneas from three-month-old female New Zealand White rabbits (*N* = 9), housed at the Weizmann Institute (Israel) at the animal facility with access to water and food as desired, were included in this study. All procedures were performed according to the Association for Research in Vision and Ophthalmology Statement for the Use of Animals in Ophthalmic and Visual Research, following the approval of the Institutional Animal Care and Use Committee.^14^ Before treatment and euthanasia, the rabbits were examined for corneal pathology by a trained cornea specialist (ALM) with a portable slit lamp (Eidolon Optical, Natick, MA, USA).

### Treatment Procedure

**Figure 1.**
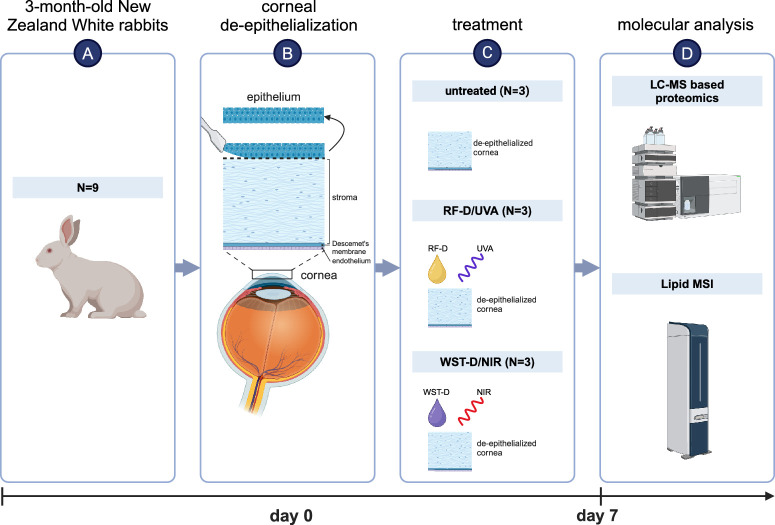
Overview of study design. Corneas from three-month-old New Zealand White rabbits (**A**, *N* = 9) were mechanically de-epithelialized (**B**) and then (**C**) left untreated (*N* = 3) or treated with either RF-D/UVA (*N* = 3) or WST-D/NIR (*N* = 3). (**D**) After one week, all rabbits were euthanized, and their corneas were analyzed by LC-MS–based proteomics and lipid MSI. Created in https://BioRender.com.

All rabbits were anesthetized by intramuscular injections of 35 mg/kg ketamine (Rhone Merieux, Lyon, France) and 5 mg/kg xylazine (Vitamed, Benyamina, Israel). The corneal epithelium of the eyes (*N* = 9) was mechanically removed with a spatula. The de-epithelialized corneas then underwent either WST-D/NIR crosslinking (*N* = 3) or RF-D/UVA crosslinking (*N* = 3) or received no further treatment (*N* = 3) (Fig. 1).

For the crosslinking treatments, a small ring of 14 mm in diameter was placed on the de-epithelialized corneas, and filled with 1 mL of WST-D or RF-D. After 20 min of impregnation with WST-D, the photosensitizer was removed by a brief rinse with 2 mL of normal saline solution. The corneas were then immediately irradiated for 30 minutes by NIR light of 753 nm at 10 mW/cm^2^ (Cerelas PDT 753; CeramOptec GmbH, Bonn, Germany). Every five minutes during irradiation, the cornea was hydrated by a drop of normal saline solution. For RF-D, after 30 minutes of impregnation, the corneas were irradiated with UVA light of 370 nm at 3 mW/cm^2^ for 30 minutes (self-constructed device of two LEDs; Roithner Lasertechnik, Vienna, Austria) and RF-D was applied every five minutes during irradiation (Dresden protocol).^6^ No WST-D, RF-D, NIR, or UVA irradiation was applied to the eyes in the control group. Prophylactic anti-bacterial Maxitrol ointment (Alcon, Geneva, Switzerland) was applied twice daily to the de-epithelialized corneas until full re-epithelialization was confirmed by slit lamp evaluation with a fluorescent dye. One week after treatment, euthanasia was initiated by intravenous injection of pentobarbital sodium (CTS Chemical Industries Ltd, Kiryat Malachi, Israel). Corneoscleral buttons were dissected, wrapped tightly in cling film and aluminum foil, flash-frozen in liquid nitrogen, and stored at −80°C until further analysis.

### Sample Preparation

All corneas were cut in half while maintaining the corneal curvature. Sagittal sections with a thickness of 20 µm were obtained using a cryostat (Leica CM1860 UV, Leica Biosystems, Wetzlar, Germany) at −23°C. For proteomics, 20 consecutive tissue sections of each cornea were collected and submerged in 5 M urea and 50 mM ammonium bicarbonate in 1.5 mL microcentrifuge tubes. Additionally, for lipid MSI and histology, three sections of each cornea treated with WST-D/NIR or RF-D/UVA crosslinking, and thaw-mounted on indium tin oxide–coated conductive glass slides (Rs 4–8 Ω/sq; Delta Technologies Ltd, Loveland, CO, USA). The untreated corneas were excluded from the lipid MSI and histology as MSI allows for the identification of regions of interest (ROI; i.e., treated and untreated parts of the corneal stroma). Here, the part of the stroma treated with either WST-D/NIR or RF-D/UVA crosslinking could be compared with the untreated stromal part as a control in the same cornea.

### Liquid Chromatography–Mass Spectrometry (LC-MS)

Cell lysis was performed by three freeze/thaw cycles between a −80°C freezer and an ultrasonic bath. The lysate was clarified by centrifugation at 15,000*g* for 30 minutes at 4°C and proteins in solution were transferred to new tubes. The samples were reduced with 20 mM dithiothreitol for 45 minutes and alkylated with 40 mM iodoacetamide for 45 minutes, followed by termination of alkylation with 20 mM dithiothreitol. Their digestion was performed with a mixture of Lys-C and trypsin (V5073; Promega Corp., Madison, WI, USA), which was added at an enzyme/protein ratio of 1:25. After two hours of digestion at 37°C in a PCMT thermoshaker (Grant Instruments, Royston, UK) at 750 rpm, the lysate was diluted with 50 mM ammonium bicarbonate and 1 M urea and further digested overnight in the thermoshaker. Digestion was terminated by adding formic acid to a 1% (v/v) concentration. The peptide samples were stored at −20°C until further analysis.

LC-MS–based, label-free quantitative proteomics was performed to identify and quantify proteins. Peptide separation was performed on a Dionex Ultimate 3000 Rapid Separation UHPLC system (Thermo Fisher Scientific, Waltham, MA, USA) equipped with a PepSep C18 analytical column (15 cm, ID 75 µm, 1.9 µm Reprosil, 120 Å). Peptide samples were first desalted on an online-installed C18 trapping column. After desalting, peptides were separated on the analytical column with a 90-minute linear gradient from 5% to 35% acetonitrile with 0.1% formic acid at a 300 nL/min flow rate.

The UHPLC system was coupled to a Q Exactive HF mass spectrometer (Thermo Fisher Scientific). Data-dependent acquisition was performed with a full MS scan from 250–1250 m/z at a resolution of 120,000 followed by MS/MS scans of the top 15 most intense ions at a resolution of 15,000.

### Data Processing

For protein identification and quantification, the data-dependent acquisition spectra were analyzed with Proteome Discoverer (version 2.2; Thermo Fisher Scientific). Within the software, the search engine Sequest was used with the SwissProt human protein database (*Homo sapiens*, SwissProt TaxID = 9606). Given the low number of reviewed protein entries (894 entries) in the New Zealand White rabbit database (*Oryctolagus cuniculus*, TaxID = 9986), the human database (20,376 entries) was used. The database search was performed with the following settings: trypsin as enzyme, two missed cleavages maximum, peptide length of at least 6, precursor mass tolerance of 10 ppm, fragment mass tolerance of 0.02 Da, dynamic modifications of methionine oxidation and protein N-terminus acetylation, static modification of cysteine carbamidomethylation. Protein quantification was performed using default label-free quantification settings. In short, for peptide abundancies, the peptide precursor intensities were used and normalization was performed on the total peptide amount. Protein ratios were calculated based on pairwise peptide ratios and a background-based ANOVA was used for hypothesis testing. Pathway enrichment analysis was performed using the online Reactome pathway database (www.reactome.org). The input data comprised a list of accession numbers of identified differentially expressed proteins. Enrichment analysis was performed for Homo sapiens pathways using a binominal test, with a significance threshold of *P* < 0.05. Multiple testing correction was applied using the Benjamini-Hochberg method.

### Lipid MSI

Slides for lipid MSI were coated with 7 mg/mL norharmane in a 2:1 chloroform/methanol solution using an automated TM-sprayer (HTX Technologies, Chapel Hill, NC, USA), with eight layers deposited at 30°C using a flow rate of 0.12 mL/min and 30 seconds’ drying time between each layer. The sections were imaged on a rapifleX MALDI-ToF/ToF instrument (Bruker Daltonik, Bremen, Germany) in positive- or negative-ion reflectron mode at 30 µm of lateral resolution. Data were acquired in a pixel-by-pixel manner by scanning the laser across a 30 × 30 µm^2^ area with 200 shots summed for each pixel in the m/z range of 200–2000. Before each measurement, red phosphorus was used for calibration. During acquisition, the data were automatically processed with TopHat baseline subtraction, Savitzky-Golay smoothing (0.01 m/z width, one cycle), and centroid peak detection (0.5 Da peak width, S/N≥5, height 78%). Acquired lipid MSI data were imported and processed using LipoStarMSI.^15^

### Hematoxylin and Eosin (H&E) Staining

After MSI, the norharmane matrix was removed and the slides were stained by hematoxylin (Merck KGaA, Darmstadt, Germany) and eosin-Y (Avantor Performance Materials B.V., Deventer, the Netherlands). Images of H&E-stained samples were acquired using a Leica Aperio CS2 image capture device (Leica, Wetzlar, Germany). The epithelium and the treated and untreated stroma were manually identified (JB) as ROI, and their borders were manually drawn using QuPath (v0.2.0, University of Edinburgh, Edinburgh, UK).^16^ Areas within the stroma without keratocytes were considered treated, because both WST-D/NIR and RF-D/UVA crosslinking are known to cause short-term depletion of keratocytes (Fig. 1).^17^ These H&E-based ROIs were co-registered with the respective lipid distribution images acquired by MSI.

## Results

### Differences in Proteome Were Observed Among Corneas Treated With RF-D/UVA and WST-D/NIR Compared to Untreated Corneas

**Figure 2.**
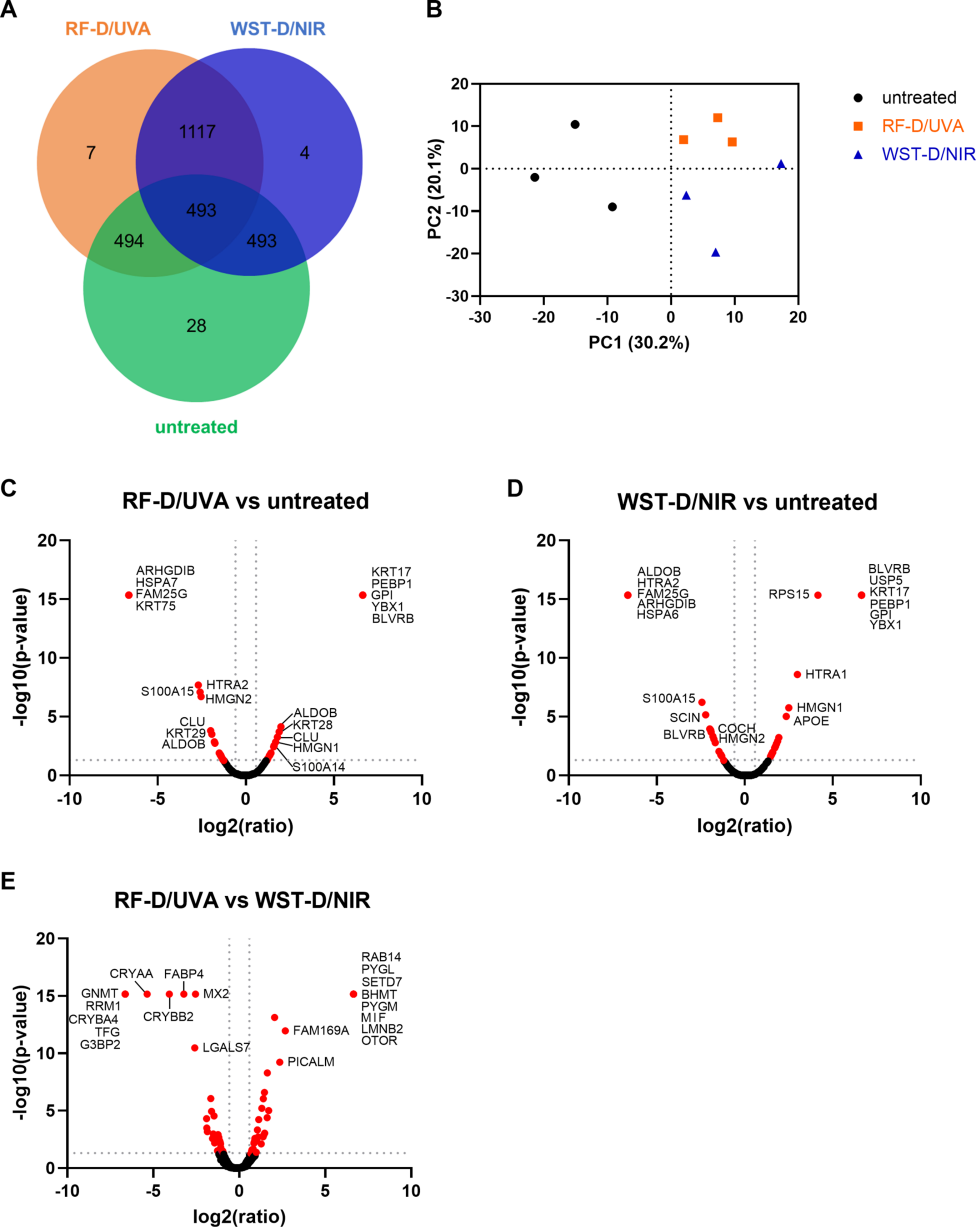
Analysis of proteins identified by LC-MS–based, label-free quantitative proteomics in RF-D/UVA–treated, WST-D/NIR–treated and untreated corneas. (**A**) Venn diagram showing the overlap of identified proteins between the RF-D/UVA–treated (*orange*), the WST-D/NIR–treated (*blue*), and the untreated group (*green*). The number of proteins that overlap between groups is indicated by the number in the overlapping areas, whereas the non-overlapping area represents the uniquely expressed proteins in that group. (**B**) PCA plot of the identified proteins shows clustering of the samples based on their proteomic profile, with the first principal component (PC1) explaining 30.2% of the variance discriminating the untreated group from both treatment groups, RF-D/UVA and WST11-D/NIR. The second principal component (PC2) explains 20.1% of the variance and separates the RF-D/UVA– and WST11-D/NIR–treated corneas. (**C–E**) Volcano plots showing both upregulated and downregulated proteins in all pairwise comparisons. The protein names are included for the top 10 most upregulated and downregulated proteins. Significantly upregulated and downregulated proteins are highlighted in *red*, defined by a log2(ratio) of ≥0.58 and ≤−0.58, respectively, and a −log10 (*P* value) ≥1.3.

A total of 1157 proteins were identified by LC-MS–based, label-free quantitative proteomics, of which 493 proteins were present in all three groups (Fig. 2A). When looking at both treatment groups, 97% of the total proteins identified (1117 out of 1157) were common, with seven and four proteins uniquely expressed in RF-D/UVA– and WST-D/NIR–treated corneas, respectively. In comparison, 28 proteins were uniquely expressed in the untreated group. The greater overlap between treatment groups was confirmed by the principal component analysis (PCA) plot, which revealed that besides the clustering in three groups, the variance between treated and untreated corneas was larger (30.2%) than the variance between both treatments (20.1%; Fig. 2B). A volcano plot showing the analysis of the effect of RF-D/UVA treatment on protein expression in the cornea demonstrated that 18 proteins were significantly upregulated and 19 were significantly downregulated, with a ≥1.5- or ≤0.67-fold difference from the untreated control (*P* < 0.05; Fig. 2C; full list in Supplementary Table S1). Among the proteins affected by WST-D/NIR treatment, 20 proteins were significantly upregulated, and 19 were significantly downregulated (Fig. 2D; full list in Supplementary Table S1). More proteins were significantly abundant in the RF-D/UVA treatment than the WST-D/NIR treatment, namely 40 proteins were significantly upregulated and 34 significantly downregulated (Fig. 2E; full list in Supplementary Table S1). Our analysis detected several inflammatory and fibrotic proteins that were differentially expressed between the treated and untreated corneas. For RF-D/UVA–treated corneas compared to untreated corneas, decorin, HtrA serine peptidase 1 (HTRA1), S100 calcium-binding protein A14 (S100A14), and clusterin were upregulated, whereas fibromodulin and hemopexin were downregulated. WST-D/NIR treatment resulted in the upregulation of S100A14, fibronectin 1, hemoglobin subunit beta, apolipoprotein E, and HTRA1 compared to the untreated controls. When comparing RF-D/UVA to WST-D/NIR treatment, macrophage migration inhibitory factor and anterior gradient protein 2 were found to be upregulated by RF-D/UVA compared to WST-D/NIR. Conversely, complement component C6, complement factor B, HTRA1, tissue inhibitor of metalloproteinases 2, serpin family A member 3, and matrix metallopeptidase 2 were upregulated in WST-D/NIR–treated corneas compared to RF-D/UVA–treated corneas. When comparing the abundance of collagen proteins, no significant difference was observed between the RF-D/UVA and WST-D/NIR treatment nor compared to untreated corneas. This suggests that although multiple proteins responded differently to the treatments, the level of collagens remained unchanged.

### Pathway Analysis of the Differentially Expressed Proteins Revealed Different Biological Processes Among Groups

**Figure 3.**
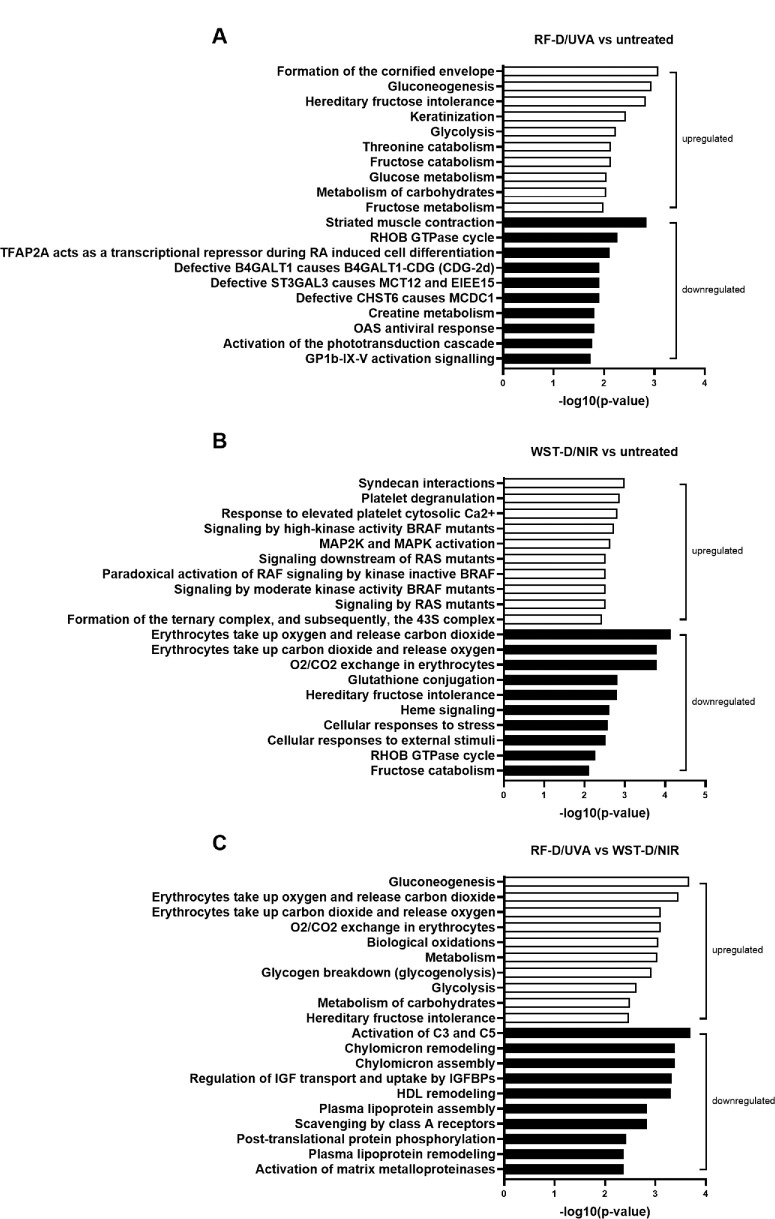
Pathway enrichment analysis reveals the 10 most significantly upregulated (*white*) and downregulated (*black*) pathways for all pairwise comparisons. The bar size represents the −log10 of the *P* value. Pathways were identified with the use of the Reactome database (reactome.org). (**A**) Upregulated pathways in the RF-D/UVA–treated group compared to the untreated group are related to terminal differentiation of keratocytes and cell metabolism. Pathways that are downregulated in the RF-D/UVA–treated group compared to the untreated group are present in numerous functional categories, including muscle contraction, signal transduction, transcription, diseases, metabolism, sensory perception, and hemostasis. (**B**) The top enriched upregulated pathway in WST-D/NIR–treated corneas compared to untreated corneas is syndecan interactions. Furthermore, there are multiple upregulated pathways involved in the MAPK signaling cascade. Additionally, pathways related to platelet response and protein synthesis are upregulated. Downregulated pathways in WST-D/NIR–treated corneas compared to untreated corneas are involved in O_2_/CO_2_ exchange in erythrocytes, heme signaling, metabolism and cellular responses to external stimuli. (**C**) RF-D/UVA–treated corneas compared to WST-D/NIR–treated corneas show upregulated pathways related to cell metabolism and O_2_/CO_2_ exchange in erythrocytes. The top enriched downregulated pathway (i.e., upregulated in the WST-D/NIR group compared to the RF-D/UVA group) is activation of C3 and C5, a central step of complement activation. Furthermore, the activation of matrix metalloproteinases is downregulated. All other downregulated pathways are related to the transport of small molecules by lipoprotein metabolism.

To gain insight into the biological processes in which the significantly differentially expressed proteins are involved, pathway enrichment analysis was performed for all pairwise comparisons, specifically for RF-D/UVA–treated corneas versus untreated corneas, WST-D/NIR–treated corneas versus untreated corneas and RF-D/UVA–treated corneas versus WST-D/NIR–treated corneas. Here, we identified the 10 most significantly upregulated and downregulated pathways (Fig. 3). The pathways significantly upregulated by RF-D/UVA treatment compared to untreated controls were related to terminal differentiation of keratocytes and cell metabolism, including gluconeogenesis, glycolysis, threonine catabolism, fructose catabolism, glucose metabolism, metabolism of carbohydrates, and fructose metabolism (Fig. 3A). The pathways significantly downregulated in RF-D/UVA–treated corneas compared to untreated corneas comprised numerous functional categories and included striated muscle contraction, RHOB GTPase cycle, repression of retinoic acid–induced cell differentiation, creatine metabolism, activation of the phototransduction cascade, GP1b-IX-V activation signaling and pathways related to disease.

The pathways significantly upregulated by WST-D/NIR treatment compared to the untreated corneas were involved in extracellular matrix (ECM) regulation and the mitogen-activated protein kinase (MAPK) signaling cascade (Fig. 3B). The most upregulated pathway was the syndecan interactions pathway, which is involved in extracellular lysyl oxidized–dependent collagen crosslinking.^18^ The significantly upregulated proteins in the syndecan interactions pathway include thrombospondin 1 and fibronectin (Supplementary Table S1). The six pathways involved in the MAPK signaling cascade include signaling by high-kinase BRAF mutants, MAP2K and MAPK activation, signaling by RAS mutants, paradoxical activation of RAF signaling by kinase inactive BRAF and signaling by moderate kinase activity BRAF mutants. Furthermore, there were two pathways related to platelet response and one pathway related to protein synthesis. The three most downregulated pathways by WST-D/NIR treatment were involved in O_2_/CO_2_ exchange. Another downregulated pathway was heme signaling, which also plays a role in the regulation of oxygen transport. Additionally, pathways related to metabolism and cellular responses to external stimuli were downregulated.

The pathways significantly upregulated by RF-D/UVA treatment compared to WST-D/NIR treatment were related to metabolism and O_2_/CO_2_ exchange in erythrocytes (Fig. 3C). When looking at significantly downregulated pathways (or upregulated in WST-D/NIR treatment compared to RF-D/UVA treatment), the top one was the activation of C3 and C5, which is involved in complement activation. Another significantly downregulated pathway was the activation of matrix metalloproteinases (MMPs) related to ECM organization, which included TIMP-2 and MMP-2 identified by proteomics (Supplementary Table S1). Such down-regulation was shown to be ROS-dependent.^19^ Other downregulated pathways were related to the transport of small molecules by lipoprotein metabolism.

### Lipid MSI Revealed Differences Between Treated and Untreated Corneas

**Figure 4.**
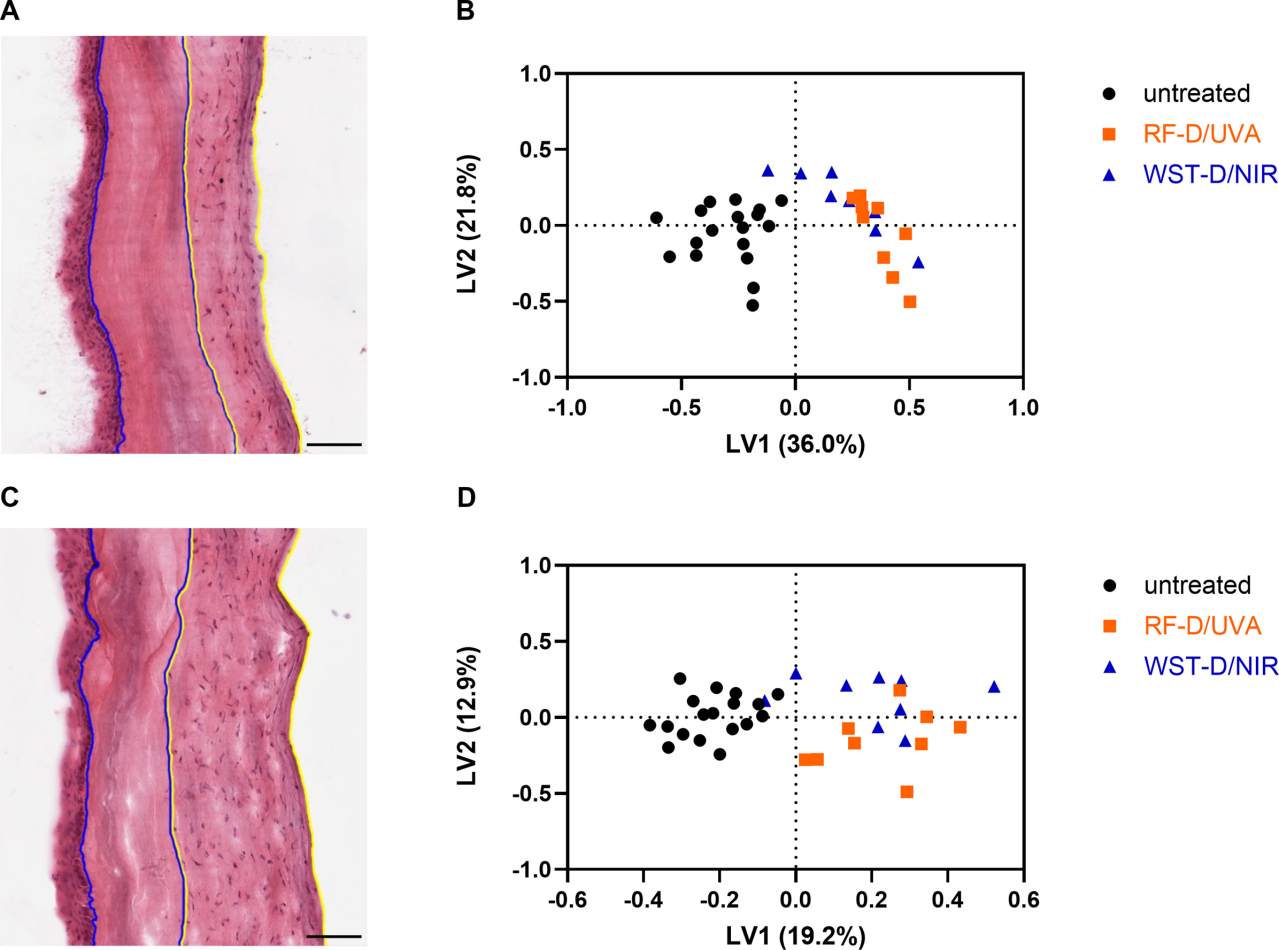
(**A, C**) Representative image of an H&E-stained section of cornea treated with RF-D/UVA (**A**) and WST-D/NIR (**C**). Based on the depletion of keratocytes after treatment, the treated (*blue*) and untreated (*yellow*) parts of the stroma were manually selected. Because lipid MSI allows for the identification of ROIs, the untreated part of the stroma was used as a control for the treated stroma from the same cornea. *Scale bar*: 100 µm. (**B, D**) Partial least squares-discriminant analysis plot of lipid profiles acquired by MSI in negative (**B**) and positive (**D**) ionization mode. The RF-D/UVA (*orange squares*) and WST11-D/NIR (*blue triangles*) treatments cluster together, showing that both groups have similar lipid profiles, whereas untreated tissue (*black circles*) harbors a different lipid profile. The first latent variable (LV1) explains 36.0% of the variance in **B** and 19.2% of the variance in **D**.

To identify the lipid profiles of treated and untreated corneal tissue, lipid MSI was performed. The untreated part of the corneal stroma was used as a control for the treated region within the same cornea. To identify the regions of interest, H&E staining was used to visualize keratocytes, which were present in the untreated stroma but depleted after treatment by RF-D/UVA or WST-D/NIR (Figs. 4A, 4C). No structural differences were observed comparing RF-D/UVA and WST-D/NIR treatments. Overall, the lipid profiles for the RF-D/UVA– and WST-D/NIR–treated stromas acquired by MSI in negative and positive ionization modes were similar (Figs. 4B, 4D). However, a clear difference in the lipid profile was observed when comparing the treated and untreated stroma. The difference was more distinct when using MSI in negative ionization mode (36.0%) compared to positive ionization mode (19.2%). Moreover, in negative ionization mode, the PCA plot (unsupervised analysis) revealed a clear separation between untreated and treated stromas, unlike MSI in positive ionization mode, whereby only the partial least squares-discriminant analysis plot showed a clear difference.

To gain insight into which specific lipids drive the different lipid profiles resulting from treatment, the 10 most significantly different lipids of the untreated and treated corneal stroma in negative (Table 1) and positive (Table 2) ionization modes were identified. For all lipids but one (m/z value of 748.5; identified as PE), LC-MS lipidomics was checked and MS2 was confirmed. The most abundant identified lipids were the phosphatidylinositols (PIs) in negative ionization mode, and the phosphatidylcholines (PCs) in positive ionization mode. The most significantly downregulated lipid in the treated compared to the untreated corneal stroma was identified as PI 18:1_20:3 (m/z 885.5) using the negative ionization mode. Moreover, the most significantly upregulated lipid was identified as PI 16:0_20:2 (m/z 861.5). When looking at the lipid MSI data using the positive ionization mode, the most significantly downregulated lipid associated with the crosslinking treatments was identified as PC 18:0_18:1 (m/z 810.6). The most significantly upregulated lipid was identified as SM 34:1;O2 (m/z 725.6). Furthermore, examination of the m/z values demonstrated a consistent −14 Da shift from m/z 720.6 (PC O-32:0) in the untreated stroma to m/z 706.5 (PC) in the treated stroma, indicating the loss of a methyl group.

**Table 1.** Negative Ionization Mode

	Untreated				Treated			
	m/z	LV1	LV2	Lipid ID	m/z	LV1	LV2	Lipid ID
1	885.5	−0.56	0.07	PI (18:1_20:3)	861.5	0.29	−0.38	PI (16:0_20:2)
2	883.5	−0.23	−0.15	PI (18:1_20:4)	835.5	0.19	0.08	PI (16:0_18:1)
3	871.5	−0.19	−0.13	PI (17:0_20:4)	742.5	0.18	−0.36	PE (18:1/18:1)
4	766.5	−0.18	−0.05	PE (18:1_20:3)	863.6	0.16	0.01	PI (18:0_18:1)
5	748.5	−0.14	−0.09	PE^*^	700.5	0.13	−0.27	PC or PE
6	899.6	−0.14	−0.09	PI (17:0_22:4)	687.5	0.13	0.18	SM 33:1;O2
7	913.6	−0.14	−0.04	PI (18:0_22:4)	833.5	0.13	−0.03	PI (16:1_18:1)
8	911.6	−0.13	−0.01	PI (18:1_22:4)	714.5	0.12	−0.09	PE (16:1_18:1)
9	887.6	−0.12	−0.46	PI (18:1_20:2)	773.5	0.09	−0.08	PG 36:2 or LBPA 36:2
10	764.5	−0.12	−0.06	PE (18:1_20:4)	786.5	0.08	−0.15	PC O-34:3

LBPA, lysobisphosphatidic acid; LV1, first latent variable; LV2, second latent variable; PC, phosphatidylcholine; PE, phosphatidylethanolamine; PG, phosphatidylglycerol; PI, phosphatidylinositol; SM, sphingomyelin.

Summary of the 10 most significantly different lipids in the untreated and treated (RF-D/UVA and WST-D/NIR combined) corneal stroma detected by lipid MSI in negative ionization mode. The table shows the m/z values, the corresponding latent variables (LV), and the lipid identifications. The lipid identifications include the lipid class, carbon chain length, and degree of saturation. When identified, the fatty acid composition of both acyl chains are provided instead of the sum composition, shown in parentheses wherein the underscore ( _ ) separates the two chains. When the stereochemical position is known, the forward slash ( / ) is used to separate the first and second chain into the sn1 and sn2 position, respectively. All lipid identifications were confirmed by MS2 fragmentation analysis, except for the lipid assignment marked with an asterisk ( ^*^ ) for which this was not possible.

**Table 2.** Positive Ionization Mode

	Untreated				Treated			
	m/z	LV1	LV2	ID	m/z	LV1	LV2	ID
1	810.6	−0.35	0.04	PC (18:0_18:1)	725.6	0.32	0.18	SM 34:1;O2
2	774.6	−0.32	−0.02	PC 35:1	732.6	0.28	0.03	PC 32:1
3	788.6	−0.27	−0.07	PC 36:1	754.5	0.25	−0.08	PC
4	796.6	−0.26	0.01	PC (17:0_18:1)	758.6	0.18	−0.27	PC 34:2
5	746.6	−0.20	−0.09	PC O-43:1	703.6	0.14	0.07	SM 34:1;O2
6	832.6	−0.17	0.05	PC (38:4)	780.6	0.14	−0.13	PC
7	720.6	−0.14	0.03	PC O-32:0	706.5	0.10	0.00	PC
8	772.6	−0.14	−0.01	PC	728.5	0.10	−0.10	PC (14:0_16:0)
9	768.6	−0.13	−0.03	PC O-34:1	740.5	0.10	−0.12	PC or PE
10	830.6	−0.11	0.04	PC (16:0_22:5)	704.5	0.09	−0.12	PC 30:1

LV1, first latent variable; LV2, second latent variable; PC, phosphatidylcholine; PE, phosphatidylethanolamine; SM, sphingomyelin.

Summary of the 10 most significantly different lipids in the untreated and treated corneal stroma detected by lipid MSI in positive ionization mode. The table shows the m/z values, the corresponding latent variables, and the lipid identifications. The lipid identifications include the lipid class, carbon chain length, and degree of unsaturation. When identified, the fatty acid composition of both acyl chains are provided instead of the sum composition, shown in parentheses wherein an underscore ( _ ) separates the two chains. All lipid identifications were confirmed by MS2 fragmentation analysis.

**Figure 5.**
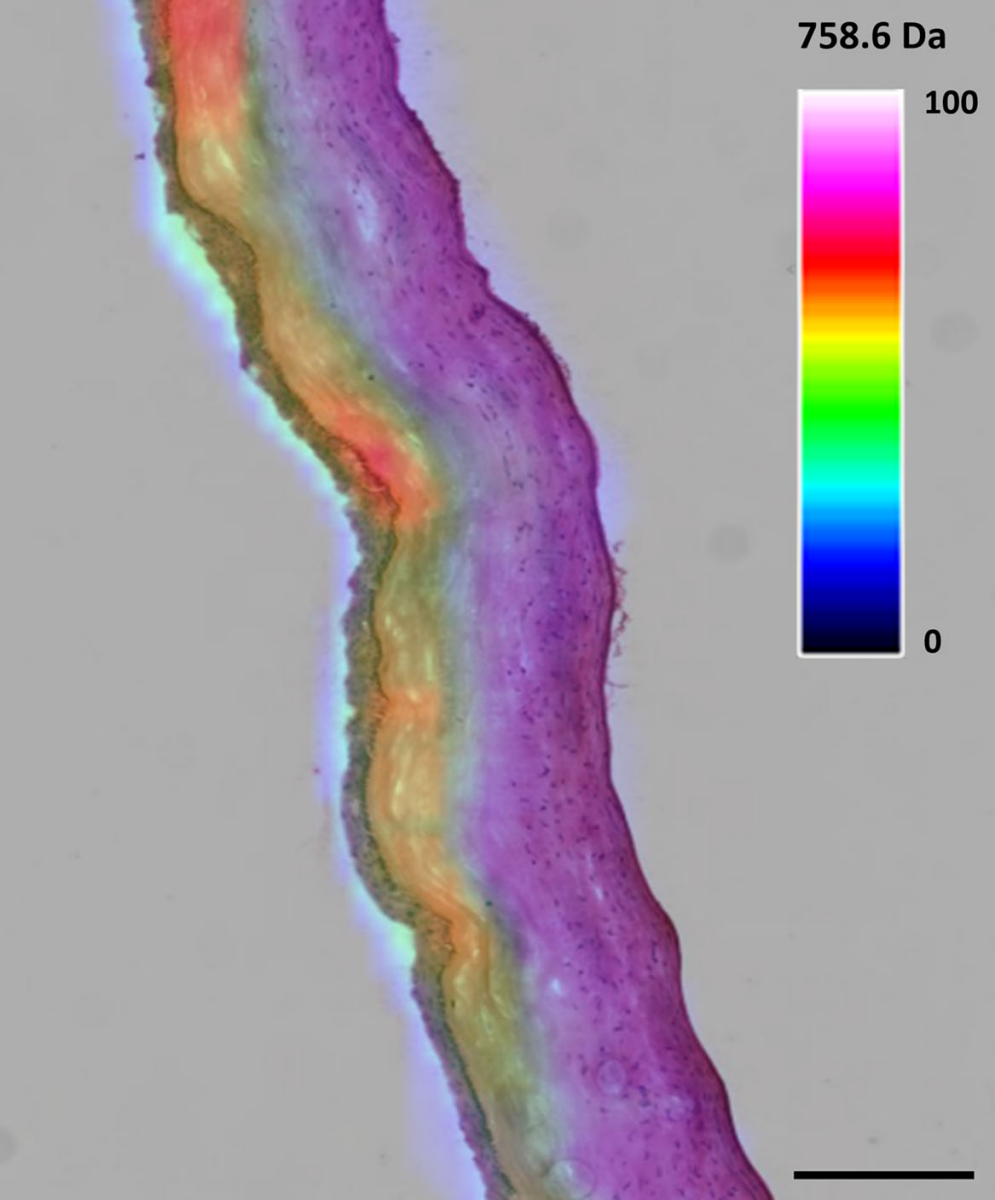
Example of H&E-stained RF-UVA–treated corneal tissue with an m/z value 758.6 Da overlay representing lipid distributions acquired in positive ionization mode. Overall, lipids were more abundant in the untreated corneal stroma, where keratocytes are present. *Scale bar*: 300 µm.

Correlating the H&E-stained sections with the m/z overlays provided additional insight into how lipidomic changes were distributed across the corneal tissue (Fig. 5). The lipids that were significantly different in the untreated group compared to both treatment groups were distributed mostly in the stromal region harboring keratocytes.

## Discussion

In this study, we performed LC-MS–based label-free quantitative proteomics and lipid MSI to identify and quantify proteins and lipids, respectively, in RF-D/UVA– or WST-D/NIR–treated rabbit corneas to elucidate the potential biological changes that occur upon corneal stiffening which may halt keratoconus progression. Our study reveals that the two treatments exhibit distinct mechanisms at the protein level, including a crucial difference in cellular metabolism, oxidative reactions, and ECM remodeling. These results contribute to the current understanding of how RF-D/UVA and WST-D/NIR treatment slow the progression of keratoconus.

Most studies aiming to elucidate the mechanisms of corneal stiffening by the application of photosensitizing agents focused on the photochemical reactions that crosslink amino residues within or between the collagen fibers of the ECM. Of particular significance and practical implication is the involvement of singlet oxygen. Early studies of RF/UVA treatment suggested that singlet oxygen generated by energy transfer from the excited RF activates the crosslinking reaction, meaning a type II photochemical reaction.^20^^,^^21^ However, it is indicated that RF/UVA treatment is oxygen-dependent and that because of the rapid oxygen consumption during UVA irradiation, this type II mechanism holds only shortly.^7^^,^^22^ There is a growing consensus that a rapidly prevailing hypoxia results in the activation of corneal crosslinking by the direct interaction of RF molecules in their excited triplet and amino acid residues of corneal proteins.^22^ Moreover, an oxygen depletion rate of two to five minutes under RF/UVA treatment has been shown, meaning corneal crosslinking predominantly happens through a type I photosensitizing mechanism by direct interaction of the RF triplet with the collagen scaffold residues.^23^ In fact, enhancing corneal crosslinking by oxygen supplementation during RF/UVA treatment or by pulsed UVA illumination to slow oxygen consumption has become a recent practice.^24^^–^^30^ So far the onset of hypoxia shortly after starting RF treatment was concluded from kinetic studies with or without additional oxygen provision. In this study, evidence for the suggested hypoxia upon RF-D/UVA treatment was provided by the upregulation of pathways related to the metabolic transformation from oxidative phosphorylation into glycolysis^31^ in RF-D/UVA–treated corneas compared to untreated corneas (Fig. 3A). Moreover, pathways significantly downregulated in RF-D/UVA–treated corneas compared to untreated corneas also indicated hypoxia after treatment, such as creatine metabolism,^32^ striated muscle contraction,^33^ RHOB GTPase cycle,^34^ repression of retinoic acid–induced cell differentiation,^35^ activation of the phototransduction cascade, GP1b-IX-V activation signaling and pathways related to disease. The upregulation of glycolysis we observed agrees with a previous study that suggested improved corneal glucose metabolism in keratoconus patients after RF/UVA crosslinking by reduced 3-OH butyric acid levels, a marker of impaired ketone body and glucose metabolism.^36^ The significantly enhanced glucose metabolism, together with the induced hypoxia, suggests that RF-D/UVA rapidly consumes the oxygen in the cornea. Therefore this research is in line with the growing body of evidence that RF/UVA treatment proceeds through singlet oxygen generation only in the first few minutes of illumination, leading to hypoxia, and thereafter triggers a metabolic shift to glycolysis.

Our proteomic analysis revealed differences in molecular responses between the RF-D/UVA and WST-D/NIR treatments. The significantly downregulated molecular pathways by WST-D/NIR treatment compared to both the RF-D/UVA treatment and the untreated controls were related to cellular metabolism and oxidative reactions (Figs. 3B, 3C). The observed difference in oxidative reactions between the RF-D/UVA and WST-D/NIR treatment is in line with the study of Marcovich et al.,^11^ which showed differences in generated ROS, wherein RF/UVA corneal stiffening involved singlet oxygen, whereas WST-D/NIR treatment generated superoxide and hydroxyl radicals. The same was observed in WST-D in solution, which also generated superoxide and hydroxyl radicals with no traces of singlet oxygen after NIR illumination.^37^ Moreover, a footprint of RF-D/UVA treatment, the fluorescence of a dityrosine bond, could not be seen after WST11/NIR treatment.^11^ This difference implies that the conditions favoring the dityrosine bond formation differ between treatments, which might suggest differences in oxidative processes or the presence of ROS. The production of singlet oxygen was suggested to be required for the initial type II photochemical mechanism at the start of RF/UVA crosslinking, as it can react further with a range of molecules inducing chemical covalent bonds between amino groups of collagen molecules and between proteoglycan core proteins.^20^^,^^21^ In contrast, the WST-D/NIR treatment is suggested to sustain normal respiration while generating hydroxyl and superoxide radicals. The constant for amino acid radical formation by hydroxyl radicals is two orders of magnitude higher than that for radical formation by singlet oxygen. Thus the maintenance of normoxia throughout WST-D treatment confirms the suggested type I photosensitizing mechanism of action and high efficiency compared with RF-D/UVA.

In contrast, lipid MSI showed similar lipid profiles for the RF-D/UVA– and WST-D/NIR–treated stromas (Figs. 4B, 4D). Nevertheless, a change in lipid profile for both RF-D/UVA– and WST-D/NIR–treated stromas compared to the untreated stroma was observed. Specifically, we found a consistent −14 Da shift in PC indicating the loss of a methyl group in the treated stroma (Table 2), which is an indication of lipid peroxidation, a process driven by ROS, which leads to oxidative modifications of lipids.^38^ Therefore we suggest that the ROS generated by the RF-D/UVA and WST-D/NIR treatment, although different, leads to oxidative degradation of lipids.

In addition to a difference in cellular respiration between both treatments, pathways related to ECM remodeling differed between RF-D/UVA and WST-D/NIR treatment. Specifically, the activation of MMPs (Fig. 3C), including TIMP-2 and MMP-2 (Supplementary Table S1), were significantly upregulated in WST-D/NIR– compared to RF-D/UVA–treated corneas. It was suggested that activation of MMP-2 is under the control of superoxide.^39^^,^^40^ Similarly, multiple studies pointed at the co-activation of TIMP and MMP-2 by superoxide radicals generated during fibrosis and tissue remodeling.^41^ This is in line with the previously mentioned generation of superoxide radicals by NIR-excited WST-D,^11^ and thus WST-D/NIR is expected to initiate a similar mechanism. MMP-2 has been thoroughly investigated in keratoconus, where its levels were found to be upregulated,^42^^–^^46^ unaltered,^44^^,^^47^^–^^49^ or downregulated.^44^^,^^50^ The high variation in reported expression levels may be due to differences in the timing of analysis, suggesting a dynamic regulation of MMPs in keratoconus and after WST-D/NIR or RF-D/UVA treatment. Multiple studies do show a consistent relative decrease in TIMP levels, including TIMP-2, compared to MMP-2 levels in corneas and keratocyte cultures derived from keratoconus patients, suggesting upregulated MMP-2 plays a role in keratoconus.^46^^,^^49^^,^^51^^–^^53^ Because TIMP-2 serves as a regulator of ECM turnover by inhibiting MMP-2, we propose that the observed upregulation of TIMP-2 was initiated as a response to upregulated MMP-2 to maintain the proper balance for corneal ECM and consequently restore corneal integrity. In line with this hypothesis, MMP-2 and TIMP-2 were identified to play a role in the remodeling of the corneal ECM during wound healing, suggesting their potential to reduce the amount of corneal damage and promote wound healing.^54^^–^^56^ This relationship suggests that WST-D/NIR treatment might have a targeted effect on the pathways involved in keratoconus, by reversing the dysregulation of MMP-2 in keratoconus by the upregulation of TIMP-2. Finally, the differences in ECM remodeling between WST-D/NIR and RF-D/UVA treatment highlight the distinct molecular mechanisms underlying their stiffening effects.

The suggestion that WST-D/NIR treatment alters specific pathways involved in ECM remodeling is supported by the observed upregulation of syndecan interactions by WST-D/NIR relative to untreated controls (Fig. 3B). Specifically, the proteins thrombospondin 1 (THBS-1) and fibronectin (FN-1; Supplementary Table S1), were upregulated after WST-D/NIR treatment compared to untreated controls. THBS-1 can interact with MMP-2 and TIMP-2, regulating their activity. FN-1 is an ECM glycoprotein that is involved in cell adhesion, migration, growth, and differentiation and can interact with collagen and proteoglycans, which might affect corneal ECM structure.^57^ Similarly, in neovascularized corneas, FN-1 was upregulated, which can be associated with changes in corneal stromal structure.^58^ Upregulation of FN-1 in both WST-D/NIR–treated and neovascularized corneas suggests its role in remodeling the ECM in response to stress or damage. Interestingly, the downregulation of THBS-1 in keratoconus patients was reported in several studies, one of which also reported downregulation of FN-1.^51^^,^^59^^,^^60^ This finding suggests that the upregulation of FN-1 and THBS-1 after WST-D/NIR treatment may counteract the ECM dysregulation observed in keratoconus. Next to the upregulation of syndecans, multiple pathways involved in the MAPK signaling cascade were upregulated in response to the WST-D/NIR treatment compared to untreated controls (Fig. 3B). The MAPK cascade functions as a central regulator in a wide range of stimulated cellular processes, involving proliferation, differentiation and development.^61^ A possible downstream effect of MAPK activation could be the identified upregulation of FN-1 and THBS-1, which can consequently affect TIMP-2 and MMP-2 expression. In line with this hypothesis, research showed that the MAPK signaling pathway can interfere with the activation of NF-kB, which can in turn regulate the expression of MMPs.^62^^–^^66^ The suggested complex interaction between MAPK, FN-1, THBS-1, MMP-2, and TIMP-2 could potentially result in ECM remodeling after WST-D/NIR treatment. The proposed ECM modulation after WST-D/NIR treatment is supported by a previous study that showed that WST-D/NIR increased resistance to enzymatic digestion, demonstrating enhanced structural integrity of the ECM.^12^ Although the concurrent upregulation of MAPK, FN-1, THBS-1, MMP-2, and TIMP-2 suggests potential involvement in ECM remodeling, further research is needed to establish their collective interaction and the underlying remodeling mechanism in the cornea.

Comparing the RF-D/UVA treatment to untreated controls, we observed a significant upregulation of pathways related to the terminal differentiation of keratocytes. We hypothesize that this upregulation might compensate for the depletion of keratocytes after the treatment by accelerating the differentiation process of the remaining progenitor keratocytes. The lack of keratocytes in the treated corneal stroma is also a highly probable reason for the observed change in lipid profile between the treated and untreated corneal stroma. Furthermore, no differences were found related to ECM remodeling, further confirming the suggested difference in the mechanism of action between RF-D/UVA and WST-D/NIR treatment. However, a study reported collagen types I and IV to be resistant to cleavage by MMP-1, -2, -9, and -13 after RF/UVA treatment of intact bovine cornea ex vivo.^66^ This observation implies that the changes in collagen structure caused by RF-D/UVA treatment protect them from degradation by MMPs. Additionally, RF-D/UVA has been found to significantly increase resistance to enzymatic digestion, similar to WST-D/NIR treatment.^12^ These findings suggest that although RF-D/UVA may not induce ECM remodeling through signaling pathways, it could provide protection by inhibiting enzymatic degradation processes, thus preserving ECM structure and arresting keratoconus progression.

In summary, this study examines the proteomic and lipidomic profiles of the cornea following the conventional RF-D/UVA and new WST-D/NIR treatment for keratoconus. Our study reveals that the two treatments exhibit distinct mechanisms, shown by significant differences in cellular respiration and ECM remodeling. Here, RF-D/UVA corneal crosslinking results in hypoxia, which induces a metabolic shift from oxidative phosphorylation into glycolysis. In contrast, WST-D/NIR treatment maintains normoxia and downregulates oxidative reactions, suggesting a more efficient treatment. Moreover, WST-D/NIR modulates specific pathways involved in ECM remodeling, which are unaffected by RF-D/UVA. This mechanistic difference could motivate the use of WST-D/NIR for tissues characterized by ECM dysregulation or other complications, particularly where oxygen enrichment is limited, such as the treatment of the posterior sclera for myopia. Although our study provides valuable insights into the biological pathways induced by both treatments, it is important to acknowledge that the study was conducted using healthy rabbits rather than a keratoconus rabbit model. Further investigations into the proteomic changes after treatment in keratoconic corneas would be interesting to investigate if the results may differ in a keratoconus-diseased state. Moreover, it would be interesting to analyze the proteomic changes after crosslinking over time to learn if the observed changes are reversible. All in all, this research adds to the current understanding of the mechanisms by which RF-D/UVA and WST-D/NIR treatment induce stiffening and slow the progression of keratoconus.

## Supplementary Material

Supplement 1
